# BRCA1 and Oxidative Stress

**DOI:** 10.3390/cancers6020771

**Published:** 2014-04-03

**Authors:** Yong Weon Yi, Hyo Jin Kang, Insoo Bae

**Affiliations:** 1 Department of Oncology, Lombardi Comprehensive Cancer Center, Georgetown University Medical Center, Washington, DC 20057, USA; E-Mails: yy226@georgetown.edu (Y.W.Y.); hk73@georgetown.edu (H.J.K.); 2 Department of Radiation Medicine, Lombardi Comprehensive Cancer Center, Georgetown University Medical Center, Washington, DC 20057, USA

**Keywords:** BRCA, oxidative stress, reactive oxygen species (ROS), carcinogenesis, detoxification

## Abstract

The breast cancer susceptibility gene 1 (BRCA1) has been well established as a tumor suppressor and functions primarily by maintaining genome integrity. Genome stability is compromised when cells are exposed to oxidative stress. Increasing evidence suggests that BRCA1 regulates oxidative stress and this may be another mechanism in preventing carcinogenesis in normal cells. Oxidative stress caused by reactive oxygen species (ROS) is implicated in carcinogenesis and is used strategically to treat human cancer. Thus, it is essential to understand the function of BRCA1 in oxidative stress regulation. In this review, we briefly summarize BRCA1’s many binding partners and mechanisms, and discuss data supporting the function of BRCA1 in oxidative stress regulation. Finally, we consider its significance in prevention and/or treatment of BRCA1-related cancers.

## 1. Introduction

**Figure 1:**
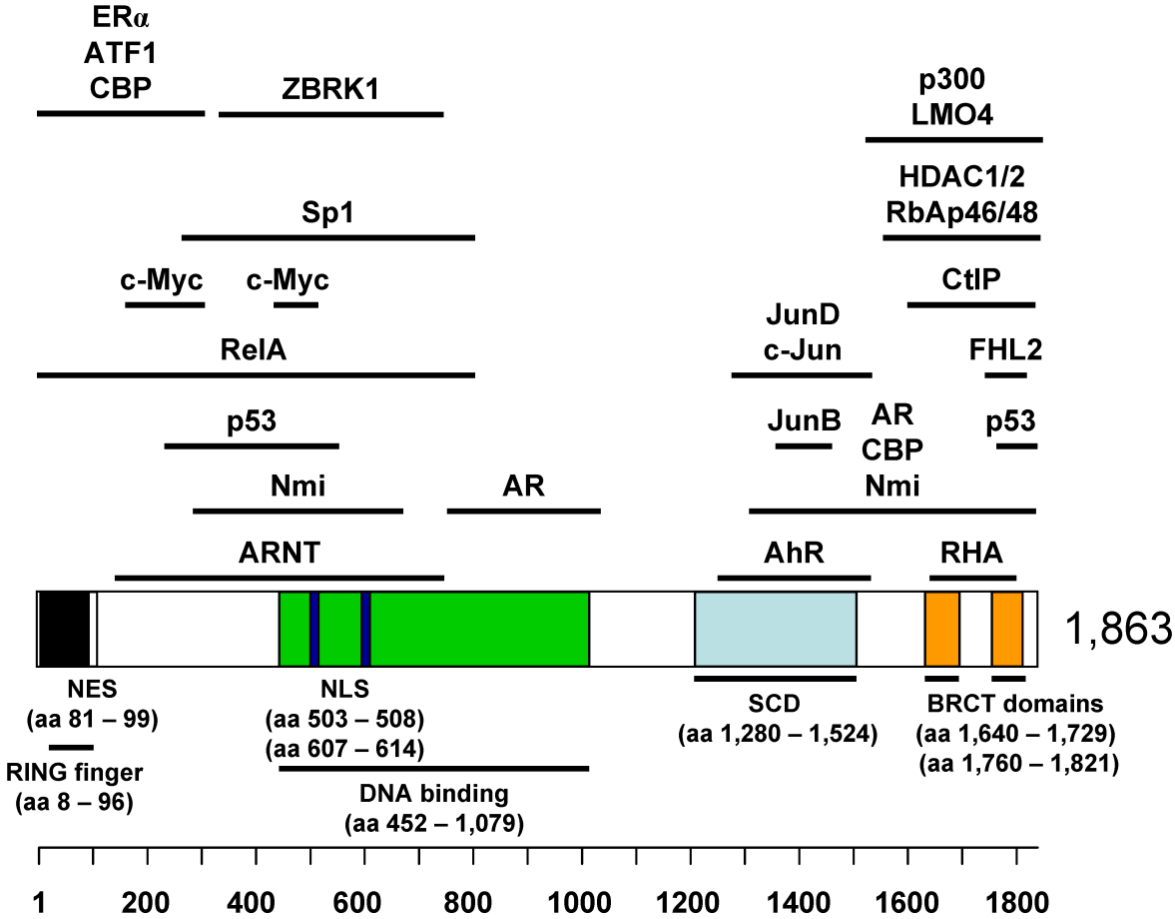
The BRCA1 protein and its binding transcription factors.

Mutations of BRCA1 are found in a high percentage of hereditary breast and ovarian cancers [1]. In the early 1990s, chromosome 17q21 was assigned to contain the locale of a gene responsible for inherited susceptibility to breast cancer in families with early-onset disease [2,3]. Subsequently, the *BRCA1* gene was cloned and analyzed to be composed of 22 coding exons distributed over ~100 kb of genomic DNA [4,5]. The full-length BRCA1 cDNA encodes a large protein of 1863 amino acids (Figure 1) [4]. The BRCA1 protein contains an *N*-terminal really interesting new gene (RING) finger domain and two BRCA1 *C*-Terminal (BRCT) domains, which are involved in various protein-protein interactions [6,7,8,9,10,11,12]. The BRCA1 protein also has a DNA-binding domain [13,14] and an SQ-cluster domain (SCD) in its central region [15]. It also has two nuclear localization signals (NLSs) [16,17] and a nuclear export signal (NES) [18]. Localization of BRCA1 is regulated by interactions with other proteins such as the BRCA1-associated RING domain protein 1 (BARD1) [19,20]. The *BRCA1* gene is conserved in mammals [21,22] and the BRCA1 protein has roles in various cellular events including cell cycle control, DNA damage signaling, maintaining genomic integrity, protein ubiquitination, and transcriptional regulation. All these responsibilities of BRCA1 may collectively participate in tumor suppression; however, precise mechanisms of how the BRCA1 protein functions as a tumor suppressor are still not yet fully understood [23,24].

Every cell is inevitably exposed to oxidative stress, either extracellular or intracellular, during its life time [25,26]. Reactive oxygen species (ROS) are produced in a controlled manner in normal cells and act as important signaling molecules to regulate various cellular functions including transcriptional regulation and signal transduction (reviewed in [25,26,27,28,29]). However, uncontrolled production of ROS causes oxidative stress resulting in DNA damage [30,31,32,33,34,35] and impaired cellular functions leading to various human diseases including cancer [26,36,37,38]. It is interesting that recent data demonstrate that BRCA1 is involved in oxidative stress regulation. These findings prompt the thought that BRCA1 might exert its tumor suppressive functions through oxidative stress regulation. Given that the mechanisms of BRCA1 tumor suppression have not yet been fully elucidated, we summarize and explore the potential role of BRCA1 in response to ROS and carcinogenesis in the context of its known functions. 

## 2. BRCA1 as a Tumor Suppressor

Women carrying germline mutations in the *BRCA1* gene have a 50%–80% lifetime risk of developing breast cancer and a 20%–40% lifetime risk of developing ovarian cancer [39]. Decrease or absence of the BRCA1 protein in sporadic breast and ovarian cancers suggests that BRCA1 functions as a tumor suppressor in nonhereditary tumors as well [40,41]. In addition, it has been reported that germline mutations in the *BRCA1* gene confer the risk of prostate [42,43,44] and pancreatic cancer [45,46,47,48,49,50,51,52,53].

Tumor suppressor functions of BRCA1 have been studied in mouse models. Deficiency of Brca1, in which *Brca1* mutations encode truncated proteins that lack the *C*-terminal half of the protein, results in early embryonic lethality ([54,55] and references therein). Mouse models carrying a series of allelic mutations in *Brca1* have been created to overcome this embryonic lethality [54]. Cells carrying these *BRCA1* mutations show genomic instability and defects in DNA damage repair [54]. However, the loss of Brca1 alone is not sufficient for tumorigenesis. Rather, multiple genetic alterations, including the inactivation of p53 and activation of a number of oncogenes caused by genomic instability in Brca1-defective cells may cause mammary tumorigenesis [54]. Most recently, two separate groups demonstrated the importance of two domains, BRCT and RING, in the tumor suppressor function of Brca1 in mouse models [56,57]. A RING mutation (C61G), found in human cancer, destabilizes the BRCA1/BARD1 heterodimer and abrogates ubiquitin ligase activity [58,59,60,61]. Mice homozygous for this mutation, in a mutated p53 background, were found to rapidly develop tumors than mice carrying a *Brca1*-null allele [56]. A mutation of S1598F in BRCT domain that ablates phosphoprotein recognition was also found to be defective in the control of genome integrity and this mutant Brca1 failed to function as a tumor suppressor in three separate mouse models of Brca1-dependent tumorigenesis [57]. Thus, genome stability is a key for healthy cells and the main function of BRCA1 is to keep the genome stable. Since ROS cause DNA damage, then it is plausible that BRCA1 normally functions to regulate oxidative stress.

### 2.1. Genome Stability, Oxidative Stress and Tumor Suppression

Genomic instability observed in BRCA1-defective cells, either by loss of function or by mutations of BRCA1, results from defects in DNA damage repair [62,63]. This genomic instability may facilitate permanent oncogenic changes in the genome [24]. Various carcinogenic insults such as ultraviolet (UV) light, ionizing radiation and environmental carcinogens generate ROS in cells. Excess ROS generate oxidative stress that sustains uncontrolled cell proliferation that eventually causes genomic instability and cancer ([30,31,32,33,34,35] and references therein). In addition, accumulating data suggest that residual oxidative stresses from xenobiotics promote tumorigenesis [64]. 

Despite evidence that maintenance of genome integrity by BRCA1 serves as an important tumor suppressor activity, the exact biochemical activity that responsible for tumor suppression by BRCA1 still remains elusive [23,24].

### 2.2. Mouse Model: Genome Stability, Oxidative Stress and Tumor Suppression

A study in *Brca1^Δ11/Δ11^p53^+/−^* mice also suggests that BRCA1 functions in the oxidative stress response. These mice display spontaneous tumor formation in mammary gland and ovary as well as lymphoma [65,66,67,68]. It has been reported that these mice exhibit increased expression of Redd1, elevated ROS and are more sensitive to oxidative stress induced lethality [69]. In this study, *Brca1^Δ11/Δ11^p53^+/−^* mice were treated with methyl-*N*-amylnitrosamine (MNAN), a carcinogen that specifically induces esophageal tumors in mice and rats [69]. In this background MNAN-treated mice had a significantly increased esophagus and forestomach tumor incidence as compared with that of control mice. Although the significance of these MNAN-induced tumors is not yet understood in human BRCA1-related cancers, these results suggest that Brca1 is involved in the regulation of ROS in carcinogenesis of the mouse.

### 2.3. Cell Studies: Genome Stability, Oxidative Stress and Tumor Suppression

In cell studies, BRCA1 was reported to associate with complex DNA damage such as double-strand breaks (DSBs) and non-DSB bistranded oxidatively induced clustered DNA lesions (OCDLs) [70,71]. In fact, a large protein complex, named BRCA1-associated genome surveillance complex (BASC), was known to be involved in the recognition of abnormal DNA structures or damaged DNA to repair them [72]. HCC1937 breast cancer cells, that harbor a 5382insC germ-line mutation of BRCA1, were demonstrated to have a significant accumulation of various types of DNA damage and chromatid breaks after ionizing radiation compared to MCF7 cells that are BRCA1 hemizygous [71]. And partial restoration of wild type BRCA1 in HCC1937 cells increased the overall repair of OCDLs [71].

BRCA1 was also reported to regulate the transcription-coupled repair of the 8-oxoguanine (8-oxoG) DNA lesion in HCC1937 cells [73]. The 8-oxoG is a well-known DNA lesion that can be caused by oxidative stress [74]. Subsequent studies have repeatedly reported that the loss of BRCA1 function results in the increase of 8-oxoG and overexpression of wild type BRCA1 increases the repair of 8-oxoG DNA lesion [75,76,77,78]. The increase of 8-oxoG DNA lesion is linked to transcriptional regulation of the base excision repair (BER) pathway by BRCA1 [76].

Besides the function of BRCA1 in BER to remove the 8-oxoG DNA lesion, varied reports suggest that BRCA1 regulates oxidative stress itself: loss of BRCA1 increases the cellular ROS and overexpression of BRCA1 suppresses ROS production [77,78,79,80,81,82]. Overexpression of wild type BRCA1 was reported to suppress both basal and the H_2_O_2_-mediated ROS production determined by carboxy-DCF fluorescence in MCF7 cells. Consistent with this, the siRNA-based BRCA1 knockdown (BRCA1-KD) enhanced both basal and the H_2_O_2_-mediated ROS production in one normal-like (MCF10A) and two breast cancer (MCF7 and T47D) cell lines [77]. Under these conditions, wild type BRCA1 reduced protein nitration and the H_2_O_2_-induced 8-oxoG formation in a prostate cancer cell line DU145. Again the 8-oxoG formation was increased in BRCA1-KD DU145 cells [77].

BRCA1-KD also enhanced the ROS accumulation in a normal-like breast epithelial cell, MCF10A in the absence or presence of benzo[a]pyrene (B[a]P) [78,79]. Under these conditions, BRCA1-KD also increased 8-oxoG formation in MCF10A cells [78]. ROS induced by multiple environmental factors such as paraquat, 2,3,7,8-tetrachlorodibenzodioxin (TCDD), 7,12-dimethylbenz[a]anthracene (DMBA), 2-hydroxyestradiol (2OHE2) and 4-hydroxyestradiol (4OHE2) were also increased in BRCA1-KD MCF10A cells in the presence of B[a]P [79]. It had also been reported that overexpression of wild type BRCA1 in HCC1937 cells reduces the generation of H_2_O_2_ during co-culture with fibroblasts [80]. The inhibition of H_2_O_2_ generation was also confirmed in an isogenic ovarian cancer cell model UWB1.289, which lacks a wild type *BRCA1* gene and UWB1.289+BRCA1, which expresses wild type BRCA1 by stable transfection [81]. More recently, it has been demonstrated that wild type BRCA1 suppresses the H_2_O_2_-induced ROS production in human aortic smooth muscle cells [82]. Thus, there is substantial evidence of oxidative stress regulation by BRCA1 in the destabilized genome.

### 2.4. BRCA1 and p53: Genome Stability, Oxidative Stress and Tumor Suppression

The tumor suppressor protein p53 also contributes to the regulation of oxidative stress in response to ROS (reviewed in [83]). BRCA1 (aa 224–550) has been found to interact with the *C*-terminus of p53 and activate its transcriptional activity at the promoter of p21 or BAX [84,85,86]. Subsequently, the *C*-terminus of BRCA1 (aa 1760–1863) has been also reported to bind p53 and is sufficient to activate the p53-dependent transcription from the p21 promoter [87]. Interestingly, BRCA1 stabilizes p53 and induces the p53-dependent transcription of genes that are involved in DNA repair and growth arrest rather than proapoptotic genes, while DNA-damaging agents-stabilized p53 leads to the transcription of multiple genes including apoptotic genes [88]. BRCA1 may also promote the accumulation of p53 in an ARF (alternate reading frame; p14^ARF^)-dependent manner: BRCA1 does not stabilize p53 in p14^ARF^-deficient A375 cells [89]. In addition, the BRCA1/BARD1 complex can stabilize p53 through phosphorylation of p53 at Ser15 residue by the ataxia telangiectasia mutated (ATM) and the ATM and Rad3-related (ATR) proteins in response to gamma- and UV-irradiation, respectively [90]. Although these results suggest that BRCA1 potentially contributes to the oxidative stress response through the regulation of p53, further experiments are needed to determine the exact role of BRCA1/p53 in this regulation. Interestingly, a recent report demonstrated that ATM kinase negatively regulates ARF by ubiquitin-dependent degradation: ATM-activated protein phosphatase 1 (PP1) antagonized Nek2-dependent phosphorylation of nucleophosmin (NPM) to liberate ARF from NPM and render it susceptible to degradation by the ULF E3-ubiquitin ligase [91]. Since stabilization of p53 by BRCA1 is dependent on both ARF and ATM, studies on the potential role of BRCA1 in ATM/ARF pathway may provide an additional insight on the regulation of p53 stability.

## 3. BRCA1 as an E3 Ubiquitin Ligase

One notable feature of BRCA1 is its E3 ligase activity that is mediated by heterodimerization with BARD1 through its *N*-terminal RING finger domain [58,59,60,61]. The ubiquitin-proteasome system is responsible for much of the regulated proteolysis in cells, and also has non-degradative regulatory functions [92]. Ubiquitin is a highly conserved 76 amino-acid protein that can be reversibly attached to other proteins by three critical enzymes: an ubiquitin-activating enzyme (E1), an ubiquitin-conjugating enzyme (E2), and an ubiquitin ligase (E3). The E3 ligase catalyzes the formation of poly- or mono-ubiquitination usually at a lysine side-chain of proteins [92]. The polyubiquitins are commonly linked through Lys48 of ubiquitin and serve as signals for proteasome-mediated proteolysis [93]. The BRCA1-BARD1 ligase complex, however, catalyzes unconventional polyubiquitins including Lys6-linked chains [94,95,96]. Although BRCA1 catalyzes polyubiquitination of numerous proteins such as the nucleophosmin/B23 (NPM1) [97], the CtBP-interacting protein (CtIP) [98], the polymerase (RNA) II (DNA directed) polypeptide H (POLR2H, also known as RPB8) [99], the transcription factor IIE (TFIIE) [100] and BRCA1 itself [95,101], there is no direct evidence that these polyubiquitinations signal degradation of substrate proteins [102,103]. A recent report demonstrated that BRCA1 polyubiquitinates G2/M cell cycle proteins including cyclin B and Cdc25C and targets these proteins to be degraded by the ubiquitin-proteasome pathway in an APC/C-independent manner in breast cancer cells [104]. BRCA1-dependent degradation of these proteins was enhanced following DNA damage, which suggests that BRCA1 targets cell cycle proteins for degradation in response to DNA damage. This may represent a possible mechanism to prevent unscheduled mitotic entry by inhibiting accumulation of these proteins [104].

BRCA1 also catalyzes the monoubiquitination of several substrates including core histones H2A, H2B, H3, H4, and the DNA-damage-responsive histone variants H2AX [60,101], as well as γ-tubulin [105] and the estrogen receptor α (ERα) [106]. Recently, it has been demonstrated that BRCA1 ubiquitinates H2A at satellite repeat DNA regions *in vivo* contributing to silencing of heterochromatin [107]. Thus, BRCA1’s E3 ubiquitin ligase activity is an important clue as to how it functions.

### 3.1. BRCA1 E3 Ligase Activity and Tumor Suppression

Homologous recombination (HR) repair is a vital process employed during stalled DNA replication or repair of DSBs caused by many DNA-damaging agents [108]. Because the BRCA1/BARD1 complex is absolutely required for HR repair, the E3 ligase activity of this complex may play a pivotal role in HR repair process (reviewed in [108,109,110]). As mentioned earlier, a RING mutation (C61G) of Brca1, which lacks ubiquitin ligase activity, failed to protect from tumor formation in mice with a mutated p53 background [56]. On the contrary, another RING mutation (I26A) that also lacks E3 ligase activity could prevent tumor formation in alternative models [57]. The exact role of the E3 ligase activity of BRCA1 in tumor suppression still remains the subject of debate [24]. 

Currently the role of the BRCA1/BARD1 ligase complex in oxidative stress is not defined yet. However, given the regulation of the nuclear factor erythroid 2-related factor 2 (NRF2) stability through physical interaction with BRCA1 (see below), it is plausible that this ligase activity contributes the modulation of proteins in NRF2 pathway.

### 3.2. BRCA1: Oxidative Stress and Ubiquitination

Interestingly, BRCA1 induces p21 expression [84,85,86,87,111], which itself exerts the antioxidant effects in response to oxidative stress through direct interaction with NRF2 [112]. NRF2 serves as a master transcription factor that activates transcription of antioxidant enzymes [113,114,115,116]. The level of NRF2 is tightly regulated by ubiquitination, which is mediated by the Kelch-like erythroid cell-derived protein with CNC homology-associated protein 1 (KEAP1) [113,114,115,116]. By binding NRF2, p21 competes with KEAP1 for NRF2 binding to inhibit KEAP1-mediated NRF2 ubiquitination and degradation [112]. Thus, p21 and indirectly BRCA1 regulate ubiquitination and degradation of NRF2 in response to oxidative stress. Indeed, the NRF2-dependent antioxidant response of p21 was further confirmed in mouse embryonic fibroblast (MEF) cells: overexpression of p21 protected Nrf2^+/+^ MEF cells but not Nrf2^−/−^ MEF cells from the H_2_O_2_-induced cytotoxicity [112].

More recently, it has been reported that BRCA1 can be physically associated with NRF2 to prevent ubiquitin-dependent degradation by KEAP1 [117]. Treatment with L-buthionine-sulfoximine (BSO) resulted in ROS accumulation and induced the interaction of overexpressed BRCA1 and NRF2 in 293FT cells [117]. Endogenous Brca1-Nrf2 interaction was also confirmed in a mouse epithelial cell line, COMMA-1D treated with BSO [117]. Mutational analysis of NRF2 revealed that an ETGE motif [118] of NRF2 is necessary to bind BRCA1 [117]. Moreover, reconstitution of wild type BRCA1 in HCC1937 cells restored NRF2 activation [117]. Since Nrf2-deficient mice exhibited a relative increase in the formation of tumor foci in the urethane-induced lung carcinogenesis model [119], loss and/or reduction of NRF2 activity by BRCA1 deficiency may contribute to the carcinogenesis of BRCA1-associated tumors. Hence, studies *in vitro* and *in vivo* demonstrate BRCA1 ability to regulate ubiquitination and degradation of proteins in response to oxidative stress.

## 4. BRCA1 as a Transcriptional Regulator

Although the BRCA1 protein has a DNA-binding domain [13,14], it is not a sequence-specific DNA-binding protein. Rather, it functions as a transcriptional modulator via physical interaction with a series of transcription factors (Figure 1) and regulates their target gene expression [120]. The potential role of BRCA1 in transcriptional regulation was first demonstrated by a fusion construct of BRCA1 *C*-terminus to the GAL4 DNA-binding domain (GAL4-BRCA1) [121,122,123]. The GAL4-BRCA1, containing a highly negatively charged C-terminus of BRCA1, could activate transcription in both yeast and mammalian cells [122]. Subsequently, the presence of certain cofactors was found to determine optimal transcriptional activation by GAL4-BRCA1 fusions [124]. In addition, this acidic *C*-terminal domain of BRCA1 was reported to induce chromatic remodeling and activation of chromosomal DNA replication when tethered to a cellular replication origin [125]. 

In addition, BRCA1 was found to be bound and co-purified with the RNA polymerase II (RNA Pol II) holoenzyme complex through an interaction with RNA helicase A (RHA) [126,127,128]. These findings suggest that BRCA1 is a component of the core transcriptional machinery. Interestingly, cancer-associated point mutations of BRCA1 disrupt the BRCA1-RNA Pol II interaction [126]. RHA and BRCA1 are known to interact with the coactivators p300 and the CREB-binding protein (CBP) [128,129]. BRCA1 is also known to modulate the phosphorylation of the *C*-terminal domain of RNA Pol II by direct interacting and inhibiting the Cdk-activating kinase (CAK) [130].

### 4.1. Binding Partners in BRCA1 Transcriptional Regulation

As mentioned, a study using GAL4-BRCA1 substantiated its role in chromatin remodeling [125]. Later, a yeast two-hybrid assay revealed the interaction of BRCA1 with the retinoblastoma (Rb)-associated protein 46 (RbAp46) [131]. RbAp46 is a growth suppressor and a component of the histone modifying and remodeling complexes. RbAp46 inhibits transactivation by a GAL4-BRCA1 fusion protein [131]. Another Rb-associated protein, RbAp48 and Rb itself were also found to interact with the BRCT domain of BRCA1 [8]. In addition, the BRCT domain interacts with proteins implicated in chromatin remodeling including the histone deacetylase (HDAC) 1 and 2 [8] or the B-related factor 1 (BRF1) subunit of the SWI/SNF-related complex [132]. The role of BRCA1 in chromatin remodeling has been shown by additional reports: BRCA1 physically interacts with the paralogous histone acetyltransferases (HATs), p300 and CBP [128,129]. These interactions enhance transcriptional activity of BRCA1 [129,132]. 

ERα, a member of the steroid hormone receptor superfamily, is activated by estrogen and plays important roles in normal development and breast tumorigenesis [133]. There are many reports demonstrating that most ERα-associated proteins participate in chromatin remodeling or in the recruitment of co-factors at the level of transcription initiation [120]. BRCA1 interacts with ERα and represses ERα-mediated transcriptional activity either in an estradiol (E2)-dependent [134,135,136] or -independent manner [137]. The ligand-dependent repression of ERα by BRCA1 is postulated to occur by binding of the N-terminus of BRCA1 (aa 1–302) to the *C*-terminal activation function (AF-2) domain of ERα [134,135]. BRCA1 may regulate the availability of p300 or cyclin D1 to compete for the AF-2 domain of ERα [138,139]. Overexpression of BRCA1 was reported to inhibit the induction of >90% of estrogen-inducible genes [140]. Given that ligand-independent repression of ERα transcription by BRCA1 was reversed by an HDAC inhibitor, trichostatin A (TSA), it is possible that HDAC activity may be involved in this regulation [137].

BRCA1 has been also known to be a transcriptional corepressor by interacting with the oncogenic transcription factor, c-Myc. BRCA1 interacts with the helix-loop-helix region of c-Myc through its two *N*-terminal regions (aa 175–303 and 443–511) and inhibits the transcriptional activity of c-Myc in the CDC25A promoter [141]. In addition, BRCA1 reverses the transforming phenotype of embryonic fibroblasts by the activation of c-Myc and Ras [141]. BRCA1 was also found to interact with the *N*-Myc-interacting protein (Nmi) through two distinct regions (aa 298–693 and 1301–1863) [142]. The formation of a ternary complex Nmi-c-Myc-BRCA1 is thought to inhibit c-Myc-induced transcription of the human telomerase reverse transcriptase (hTERT) promoter [142]. More recently, a number of genes have been identified as downregulated targets of BRCA1 through its interaction with c-Myc [143].

BRCA1 was also found to interact with CtIP [8,9,10,11,12]. The CtIP is an interacting partner of the transcriptional repressor CtBP (carboxyl-terminal binding protein) and is implicated in the repression of gene transcription [144]. The *C*-terminal region (aa 1602–1863) of BRCA1 was identified as a binding region to CtIP [8,9,10,11,12]. One proposed model is that phosphorylation of BRCA1 and CtIP by ATM following DNA damage results in dissociation of BRCA1 and CtIP and BRCA1-mediated upregulation of the growth arrest and DNA-damage-inducible gene 45α (GADD45α) protein [145]. However, dissociation of BRCA1-CtIP complex by DNA damage remains controversial [12,146].

The zinc finger and BRCA1-interacting protein with a KRAB domain 1 (ZBRK1) is a sequence-specific transcriptional corepressor, which binds to the GADD45α intron 3 as a complex with BRCA1 [147]. The *C*-terminal repression domain of ZBRK1 functions in a BRCA1-, HDAC-, and promoter-specific manner, while the *N*-terminal KRAB repression domain of ZBRK1 exhibits no BRCA1 dependency and broad promoter specificity [148]. Additionally, the angiopoietin-1 (ANG1) and the high mobility group AT-hook 2 (HMGA2) have been identified as targets of the ZBRK1/CtIP/BRCA1 repressor complex [149,150]. The derepression of transcriptional repression of these genes by removing the ZBRK1/CtIP/BRCA1 repressor complex has been reported to enhance mammary tumor growth or tumorigenesis [149,150]. BRCA1 also induces GADD45α transcription through interaction with transcription factors such as the octamer-binding transcription factor-1 (OCT-1) and the nuclear transcription factor-Yα (NF-YA) [151].

BRCA1 has been found to induce p21 in response to interferon gamma (IFNγ) through its interaction with the signal transducer and activator of transcription 1 (STAT1) [111]. Many, not all, of the IFNγ-responsive genes were identified as targets of BRCA1 and the induction of some of them was synergistically enhanced by IFNγ [152]. Among them, the interferon regulator factor 7 (IRF7) is a key molecule in the interferon signaling in response to viral infection [153]. BRCA1 was also found to bind p65/RelA subunit of the nuclear factor-κB (NF-κB) and stimulate the tumor necrosis factor-α (TNF-α) and the interleukin-1β (IL-1β)-induced transcription of NF-κB-target gene promoters [154]. It has been also reported that the interaction of BRCA1 and NF-κB is necessary to efficiently activate NF-κB activity after camptothecin treatment [155]. NF-κB was also known to constitute a complex with BRCA1-CtIP to regulate DNA DSB repair [156]. These results suggest another role of BRCA1 in the immune response to viral infection [120] as well as tumor immune surveillance [136].

It has been also reported that BRCA1 binds and stabilizes the hypoxia-inducible factor-1α (HIF-1α) and activates its target gene vascular endothelial growth factor (VEGF) [157]. Under hypoxic condition, BRCA1 was recruited to the VEGF promoter along with HIF-1α. Consistent with this, siRNA-based BRCA1-KD reduced the VEGF secretion in cultured cells following hypoxia [157]. BRCA1 was also known to repress estrogen-induced VEGF expression and secretion through interaction with ERα [158].

Expression and activation of the insulin-like growth factor-1 receptor (IGF-1R) is commonly detected in various human cancers [133,159,160,161]. The activated IGF-1R has been reported to be associated with poor survival of breast cancer patients [161]. Overexpression of wild type BRCA1 suppressed the IGF-1R promoter activity in various cell lines and reduced the endogenous levels of IGF-1R mRNA [162]. The *N*-terminal region of BRCA1 (aa 260–802) was reported to bind Sp1 transcription factor that strongly stimulates IGF-1R transcription [163]. Electrophoretic mobility shift assays (EMSAs) demonstrated that BRCA1-Sp1 interaction reduced the DNA-binding of Sp1 in a dose-dependent manner [163]. More recently, it has been reported that BRCA1 negatively regulates IGF-1 expression through an estrogen responsive element-like (EREL) site of IGF-1 promoter in an estradiol-dependent manner [164].

BRCA1 also modulates transcriptional activity of diverse transcription factors including the activating transcription factor 1 (ATF1) [165], the four and a half LIM only protein 2 (FHL2) [166], and so on. The significance of these regulations in the tumor suppressor function of BRCA1 remains to be determined. In the long list of BRCA1-interacting transcription factors (Table 1), several proteins, such as AhR/ARNT and NRF2, actively regulate the transcription of various genes in the oxidative stress response. The interactions of BRCA1 with these transcription factors in response to oxidative stress are discussed in following section.

**Table 1:** Transcription factors that bind to BRCA1.

Transcription factor	Region in BRCA1 (aa)	Assays	References
AhR	1241–1530	Co-IP/GST	[167]
AR (androgen receptor)	758–1064 & 1314–1863	GST/M2H	[168]
ARNT	131–757	Co-IP/GST/GAL4	[169]
ATF1	1–304	Co-IP/GST/Y2H/GAL4	[165]
BRF1	N/D	AP/GST	[132]
CBP	1–300 & 1314–1863	GST	[129]
CtIP	1602–1863	Co-IP/GST/Y2H	[10]
E2F1	N/D	GST	[170]
E2F4	N/D	GST	[170]
ERα	1–300	Co-IP/GST	[135]
FHL2	1756–1852	GST/GAL4/Y2H	[166]
HDAC1	1553–1863	GST	[8]
HDAC2	1553–1863	GST	[8]
HIF-1α	N/D	Co-IP	[157]
JunB	1343–1440	Co-IP/GST/GAL4/Y2H	[171]
JunD	AD1	Co-IP/GST/GAL4/Y2H	[171]
c-Jun	AD1	Co-IP/GST/GAL4	[171]
LMO4	1528–1863	Co-IP/GST/Y2H	[172]
c-Myc	175–303 & 443–511	Co-IP/GST/Y2H	[141]
NF-YA	N/D	Co-IP	[151]
Nmi	298–693 & 1301–1863	Co-IP/GST/Y2H	[142]
NRF2	N/D	Co-IP	[117]
OCT-1	N/D	Co-IP	[151]
p300	1528–1863	Co-IP/GAL4	[129]
p53	224–550 & 1760–1863	Co-IP/GST	[86,87]
RbAp46	1553–1863	Co-IP/GST/GAL4/Y2H	[8,131]
RbAp48	1553–1863	Co-IP/GST	[8]
RelA	1–802	Co-IP/GST	[154]
RHA	1650–1800	GST/GAL4/Y2H	[128]
Sp1	260–802	Co-IP/GST	[163]
STAT1	N/D	Co-IP/GST/M2H	[111]
STAT5A	N/D	Co-IP	[173]
USF2	N/D	Co-IP	[174]
ZBRK1	341–748	Co-IP/GST/Y2H	[147]

Abbreviations, AP: affinity purification; Co-IP: co-immunoprecipitation; GAL4: GAL4 reporter gene assay; GST: GST pull-down; M2H: mammalian two hybrid; N/D: not determined; Y2H: yeast two hybrid.

### 4.2. BRCA1 Regulation of Oxidative Stress through Transcriptional Activities

In addition to all the functions of BRCA1, it regulates the expression of detoxification enzymes including the UDP-glucuronosyltransferase isoform 1A1 (UGT1A1), UGT1A9, and NRF2 through transcriptional regulation by binding to their promoter regions in response to B[a]P [175]. All these genes contain xenobiotic responsive elements (XREs) in their promoter regions and are known to be regulated by a transcription factor aryl hydrocarbon receptor (AhR) [175]. Induction of NRF2 by AhR was also reported previously [176]. The AhR is a major transcriptional activator in xenobiotic responses. Upon ligand binding, AhR forms a complex with the AhR nuclear translocator (ARNT) to translocate into the nucleus and activate the transcription of its target genes [177]. BRCA1 is known to bind AhR and ARNT to activate the transcription of their target genes in breast cancer cells [167,169]. The *N*-terminal region of BRCA1 (aa 131–757) may be sufficient to interact with ARNT [169] and the *C*-terminal region (aa 1241–1530) containing the activation domain 1 (AD1) of BRCA1 has been also found to interact with AhR [167]. Interaction between BRCA1 and AhR was enhanced by xenobiotic TCDD in breast cancer cells [167] and BRCA1 was recruited to the promoter regions of CYP1A1 and CYP1B1 along with ARNT/AhR following TCDD exposure [169]. It is also reported that BRCA1 is recruited to the promoter regions of UGT1A1, UGT1A9 and NRF2 in human normal-like breast MCF10A cells after B[a]P treatment [175]. Surprisingly, BRCA1-KD reduced the binding of ARNT to XRE regions of these promoters in chromatin immunoprecipitation assays. As expected, wild type BRCA1 further activated the XRE-dependent transcriptional activation in the presence of B[a]P in MCF10A cells. Consistently, BRCA1-KD abolished the B[a]P-induced transcriptional activity of UGT1A1 and NRF2 promoters in MCF10A cells [175]. 

Systematic comparisons of gene expression profiles have revealed that BRCA1 regulates expression of a wide range of genes in human prostate (DU-145) and breast (MCF7) cancer cells [178,179]. Importantly, overexpression of BRCA1, in either DU-145 or MCF7 cells, up-regulates several NRF2-target genes including NQO1, MGST1/2, GSTA2, G6PC, ME2, *etc.* [179]. Consistently, antioxidant response genes were downregulated in Brca1-deficient MEF cell lines [179]. As a transcription factor, NRF2 binds to a specific DNA sequence known as antioxidant response element (ARE) in the promoter regions of its target genes and activates transcription of these genes [180]. Interestingly, reporter gene assays utilized with an ARE-luciferase (Luc) construct demonstrated that overexpression of wild type BRCA1 enhanced NRF2-dependent transcription [179] and BRCA1-KD by siRNA reduced the NRF2-dependnet transcription in various cell lines [78,179]. Reporter gene assays with expression of different BRCA1 mutants suggest that the *N*-terminal domain of BRCA1 is necessary and sufficient to stimulate NRF2-dependent transcriptional activation of the ARE-Luc reporter [179]. Overexpression of wild type BRCA1 conferred resistance of DU-145 and Brca1-defective MEF cells to oxidative stress-inducing agents (H_2_O_2_ and paraquat); while BRCA1-KD sensitized DU-145 cells to these agents in MTT cell viability assay [179]. Wild type BRCA1 also attenuated the loss of glutathione (GSH) in response to H_2_O_2_ in human cancer cell lines, DU-145, LNCaP and MCF7 [179]. Interestingly, a recent study reported that NRF2 induced the transcription of the *BRCA1* gene through binding to an ARE in the *BRCA1* gene promoter [181].

## 5. BRCA1, an ROS Sensor?

Despite all these results supporting the role of BRCA1 in the oxidative stress response, the mechanism of ROS sensing by BRCA1 still remains to be determined. Since BRCA1 is extensively phosphorylated at multiple residues by various kinases including ATM, ATR, the DNA-dependent protein kinase (DNA-PK), the checkpoint kinase 2 (CHK2), the cyclin-dependent kinases (CDKs), the aurora kinase A (AURK), and AKT (reviewed in [62,182,183,184]), it is possible that these multiple phosphorylations may activate BRCA1 function in response to ROS. In fact, recent evidence supports that oxidative stress activates diverse protein kinases including ATM, ATR, and DNA-PK ([185,186,187,188,189] and reviewed in [25,26,27]). In addition, oxidative stress triggers various post-translation modifications of proteins such as methylation, acetylation, sumoylation, nitrosylation, nitration, glutathionylation, and oxidation [190,191,192]. Thus, it stands to reason that BRCA1 and its associated kinases sense oxidative stress and activate tumor suppression activities. Future studies of BRCA1 modifications by oxidative stress are likely to help our understanding of BRCA1 function in the oxidative stress response and tumor suppression. 

## 6. Conclusions

**Figure 2:**
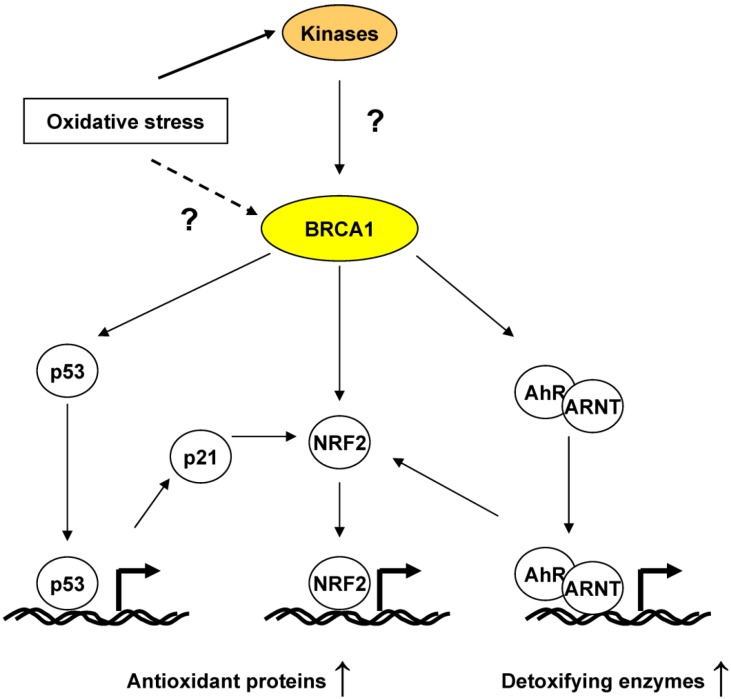
BRCA1 in the oxidative stress response.

Regulation of oxidative stress by BRCA1 has been less recognized than its other functional aspects. However, there is considerable mounting evidence suggesting that BRCA1 actively regulates ROS in response to oxidative stress at multiple levels (Figure 2). Since oxidative stress causes carcinogenesis through genomic instability by way of DNA-damage and uncontrolled cell proliferation, regulation of the oxidative stress response by BRCA1 may constitute a wheel of the chariot of the BRCA1 tumor suppression. 

ROS have the Janus faces in human cancers: uncontrolled ROS accelerate carcinogenesis in normal cells; and increased ROS may directly induce apoptotic death of cancer cells [30,193]. Consistently, antioxidant phytochemicals can be chemopreventive in early stage of carcinogenesis and can protect tumor cells from oxidative injuries [34,193]. Recently, targeting ROS has been revisited as a general therapeutic approach to treat cancers [193,194,195,196]. As an example, piperlongumine selectively induces the ROS level and apoptosis in cancer cells, but not in normal cells [197]. In fact, many conventional cancer therapies, either chemotherapeutics or radiotherapy, induce ROS in cancer cells [193,194,195,196]. Future studies will be focused on how specifically induce ROS in different types of cancers.

Taken together, the emerging role of BRCA1 as a sensor and regulator of ROS has potentially important implications to prevent and/or treat BRCA1-associated tumors. As shown in our recent study [79], surveillance of environmental factors in the context of the oxidative stress (or ROS production) in BRCA1-defective cells may provide a valuable tool to assess the risks of various environmental factors that can potentially induce BRCA1-associated carcinogenesis. This approach will be more helpful to determine potential carcinogens from the variety of environmental factors in conjunction with Brca1 mouse models, which spontaneously develop Brca1-associated mammary gland tumors [65,66,67,68]. In addition, this approach can be also applied to identify potential chemopreventive agents. For example, antioxidant phytochemicals such as sulforaphane or resveratrol can be helpful to prevent breast or ovarian cancers that carrying BRCA1 mutations by removing ROS. On the other hand, increasing cellular ROS production may be utilized to treat BRCA1-associated cancers. Indeed, BRCA1 mutated cancers are particularly sensitive to genotoxic and oxidative agents including PARP inhibitors and chemotherapeutics [198,199,200,201]. Thus, specific targeting of oxidative stress in BRCA1-defective tumors may provide alternative treatment options [202,203].
